# Effect of alteplase, benzodiazepines and beta-blocker on post-stroke pneumonia: Exploration of VISTA-Acute

**DOI:** 10.1371/journal.pone.0281617

**Published:** 2023-05-01

**Authors:** Thanh G. Phan, Richard Beare, Philip M. Bath, Svitlana Ievlieva, Stella Ho, John Ly, Amanda G. Thrift, Velandai K. Srikanth, Henry Ma, on behalf of the VISTA-Acute Collaborators

**Affiliations:** 1 Department of Neurology, Monash Medical Centre, Clayton, Australia; 2 Stroke and Aging Research Group, School of Clinical Sciences at Monash Health, Monash University, Melbourne, Australia; 3 Department of Medicine, Peninsula Health and Central Clinical School, Monash University and National Centre for Healthy Ageing, Melbourne, Australia; 4 Murdoch Children Institute of Research, Melbourne, Australia; 5 Division of Clinical Neuroscience, Stroke Trials Unit, University of Nottingham, Nottingham, United Kingdom; 6 Department of Pharmacy, Monash Health, Clayton, Australia; Universita degli Studi di Roma La Sapienza, ITALY

## Abstract

**Background:**

Post-stroke pneumonia is a frequent complication of stroke and is associated with high mortality. Investigators have described its associations with beta-blocker. However, there has been no evaluation of the role of recombinant tissue plasminogen activator (RTPA). We postulate that RTPA may modify the effect of stroke on pneumonia by reducing stroke disability. We explore this using data from neuroprotection trials in Virtual International Stroke Trials Archive (VISTA)-Acute.

**Method:**

We evaluated the impact of RTPA and other medications in random forest model. Random forest is a type of supervised ensemble tree-based machine learning method. We used the standard approach for performing random forest and partitioned the data into training (70%) and validation (30%) sets. This action enabled to the model developed on training data to be evaluated in the validation data. We borrowed idea from Coalition Game Theory on fair distribution of marginal profit (Shapley value) to determine proportional contribution of a covariate to the model. Consistent with other analysis using the VISTA-Acute data, the diagnosis of post-stroke pneumonia was based on reports of serious adverse events.

**Results:**

The overall frequency of pneumonia was 10.9% (614/5652). It was present in 11.5% of the RTPA (270/2358) and 10.4% (344/3295) of the no RTPA groups. There was significant (p<0.05) imbalance in covariates (age, baseline National Institutes of Health Stroke Scale (NIHSS), diabetes, and sex). The AUC for training data was 0.70 (95% CI 0.65–0.76), validation data was 0.67 (95% CI 0.62–0.73). The Shapley value shows that baseline NIHSS (≥10) and age (≥80) made the largest contribution to the model of pneumonia while absence of benzodiazepine may protect against pneumonia. RTPA and beta-blocker had very low effect on frequency of pneumonia.

**Conclusion:**

In this cohort pneumonia was strongly associated with stroke severity and age whereas RTPA had a much lower effect. An intriguing finding is a possible association between benzodiazepine and pneumonia but this requires further evaluation.

## Introduction

Stroke is a leading cause of disability worldwide and results in significant economic and societal cost [1]. Patients who have post-stroke pneumonia have higher mortality and disability than those patients without this complication [2]. Investigators have reported a 10% prevalence of post-stroke pneumonia [2]. The frequency of pneumonia is higher among patients with low level consciousness or among those admitted to intensive care [3]. Several different approaches have been employed in trial settings to prevent post-stroke pneumonia. These approaches, such as prophylactic administration of antibiotics and use of nasogastric tube feeding, have not been successful [2,4–6]. There is a suggestion that metoclopramide may reduce the risk of post-stroke pneumonia among patients who are fed using a nasogastric tube but such finding was confined to one report [7].

Using data from from Virtual International Stroke Trials Archive (VISTA)-Acute, investigators have reported associations between beta-blockers, but not benzodiazepines, and post-stroke pneumonia [8,9]. Beta-blockers had been postulated to have an effect by dampening activation of the sympathetic pathway. The sympathetic pathway has been postulated to be activated as part of immune dysfunction stroke [10]. Recombinant tissue plasminogen activator (RTPA) on pneumonia has been shown to reduce stroke severity. The strong relationship between stroke severity and pneumonia prompted us to consider the possibility that reversal of stroke severity such as by RTPA can decrease the risk of pneumonia. In our previous analysis of data from a single centre, we had observed only 4/85 (4.7%) cases of pneumonia among patients who received RTPA compared with higher frequency of pneumonia (6.6%) in the entire cohort [11]. In the larger VISTA-Acute cohort, we aimed to evaluate the effect of RTPA on development of post-stroke pneumonia taking advantage of information available in this archive on different medications such as sleeping medications, antipsychotic drugs [8,9]. These medications were included as they are centrally acting and can impact on level of consciousness. We explore a model of post-stroke pneumonia using the new development of interpretable machine learning to make the findings from random forest accessible [12–14].

## Method

We used data from the Virtual International Stroke Trials Archive (VISTA)-Acute archives of stroke clinical trials [15]. This data pertains to neuroprotection trials rather than recent RTPA or thrombectomy trials. The VISTA-Acute data were pooled from de-identified randomized control trials. The governance of the registry can be found at https://www.virtualtrialsarchives.org/vista/. The analysis of the results of this study was performed on virtual network computer provided by VISTA-Acute. As such the authors cannot provide the data. However, the data used in this study is available on request from VISTA-Acute, who owns the data. The authors confirm that others would be able to access these data in the same manner. The authors did not have any special access privileges that others would not have.

The following terms were used in the search strategy of this dataset: imaging data; National Institutes of Health Stroke Scale (NIHSS) on admission and at 24 hours; physiological variables (systolic blood pressure, blood glucose level); demographic data (age, sex); stroke risk factors; thrombolysis treatment with recombinant tissue plasminogen activator (RTPA); -Alberta Stroke Program Early CT Score (ASPECTS) score on CT scan [the ASPECTS assesses the extent of ischemia over 10 regions of the middle cerebral artery territory] [16]; medication data (drug class such as beta-blockers (BB2), antipsychotics (PSYCH2), benzodiazepines (BENZ2), sleeping aid medications (SLEE2)); clinical data (atrial fibrillation (AFIB), heart failure (CHF), ischemic heart disease (IHD), diabetes, hypertension); and modified Rankin outcome within 90 days of stroke. A Rankin score of 0 signifies no symptom while 6 signifies death. In this study disability is defined by a Rankin score >2.

Consistent with previous works from VISTA-Acute on this subject, diagnosis of post-stroke pneumonia was based on reports of serious adverse event within 10 days of onset [8]. We had used a broad collection of terms to assign the diagnosis of pneumonia. These terms include bronchopneumonia but not upper tract infection. We evaluate medications which the patients are taking. We extract the drug class from the list of medications that the patient was on. This data is part of the metadata acquired during drug trial. These drugs are stored with their Anatomical Therapeutic Chemical Classification System (ATC code) within VISTA. These drugs such as beta-blockers were not the the subject of the trial but were drugs prescribed by clinicians in daily management. VISTA-Acute does not include data on the type of dysphagia screen used, details about mechanical ventilation nor evaluation of immune dysfunction.

Steps in machine learning to identifed features important to post-stroke pneumonia is listed below.

1-Search text for terms to diagnose pneumonia
2-Partition data to training and validation
3-Perform Random Forest on training dataset
4-Evaluate the model again on validation dataset
5-Asssess importance of identified features using Shapley value

### Statistical analysis- machine learning

**Fig 1:**
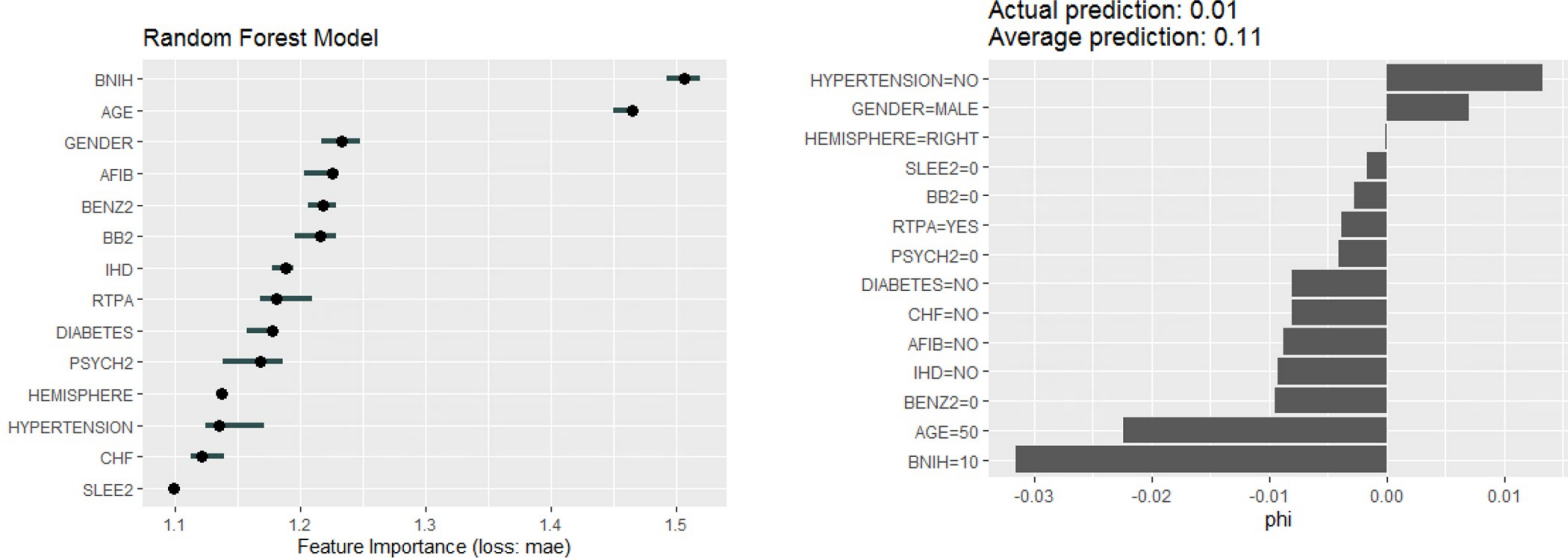
Shapley value (phi) and feature importance provide different views on the model. Shapley value illustrates the ‘payout’ of the feature within the model and the direction of its impact on the classification of pneumonia. The difference between the actual and average prediction implies the sum of the payout for prediction is 0.10. Benzodiazephine has a larger payout that alteplase (RTPA) or beta-blockers on pneumonia. Feature importance illustrates the impact on the model’s error by permuting the features. BNIH = baseline NIHSS, BENZ2 = benzodiazepine, BB2 = beta blockers, SLEE2 = non-benzodiazepine sleeping medications, IHD = ischemic heart disease, CHF, congestive heart failure, AFIB = atrial fibrillation, RTPA = recombinant tissue plasminogen activator.

**Fig 2:**
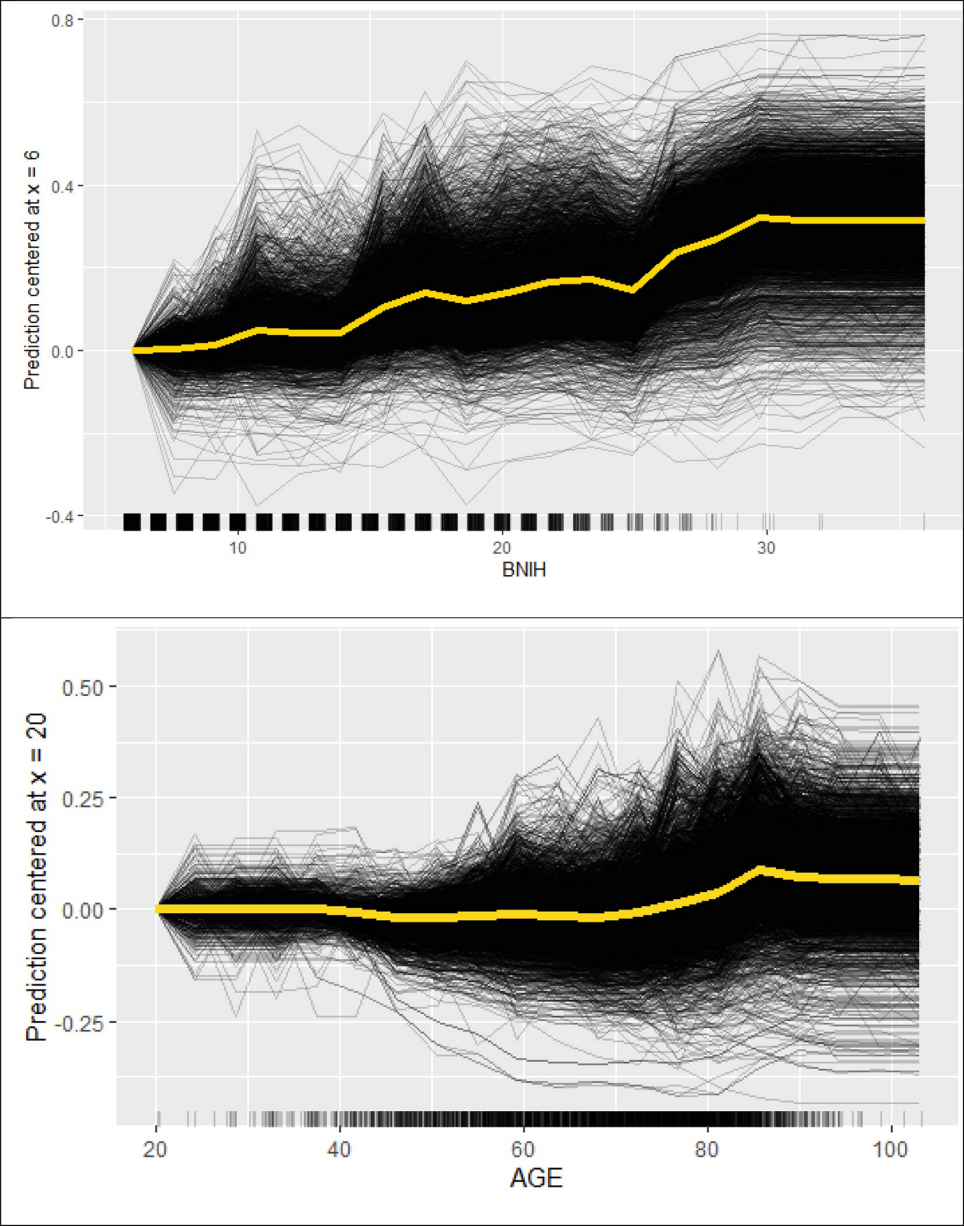
The lines represents the prediction of pneumonia each patient’s baseline NIHSS. The yellow line is the average of the lines for all patients. The dense black squares on the x-axis represent regions with data and regions from high 20s onward indicate that the data is less strong. The probability of pneumonia increased from tne age of 80 on wards.

We used random forest, a supervised machine learning method related to decision tree analysis, which employed random selection of covariates and patients from the dataset to create multiple trees (S1 Fig). This form of ensemble learning utilises the average outcome of multiple trees (n = 200) or ‘wisdom of the crowd’ to create the final model. The analysis was performed using *randomForest* package in R statistical programming environment. Given the interest in finding out the covariates making largest contribution to pneumonia, we calculated the marginal contribution of each covariate to the model or Shapley value (phi) [13,17]. The Shapley value analysis is based on the Nobel prize winning works (Economics) by Lloyd Shapley on Coalition (Cooperative) Game Theory. The marginal contribution is determined as the average of all permutations of the coalition of the covariates containing the covariate of interest minus the coalition without the covariate of interest. We used the feature importance analyses to assess the impact of permuting (re-ordering) the feature (covariate) on the model’s prediction error (Fig 1). The individual conditional expectation (ICE) curve describes the effect of the individual observation (e.g. baseline NIHSS) on the outcome (probability of pneumonia, Fig 2) [18]. Next the interaction strength of the feature is measured using H-statistic (see S2 Fig for example of interaction strength with covariate) [12]. The measure how much of the variation of the prediction of the feature depends on the interaction with other features. The H-statistic is 0 if there is no interaction and 1 if the effect of the prediction comes from the interaction. This analysis was performed using *iml* package in R [14].

We used the standard approach in machine learning, partitioning the data into training (70%) and validation (30%) sets. The model was developed using the training sample, and then the very same model is applied to the validation sample to evaluate if the model achieves the same discrimination result. We test the model in several ways including measuring the AUC and the out of bag error. The discrimination of the model is measured by area under Receiver Operating Characteristics (ROC) curve (AUC). An AUC of 0.50–0.59 indicates that it is no better than chance, 0.60–0.69 indicates poor discrimination, 0.70–0.79 indicates fair discrimination; 0.80–0.89 indicates good discrimination and >0.90 indicates excellent discrimination.

## Results

In total, 5653 eligible patients were identified, with 3087 (54.6%) male, median age 71 years (IQR 54–88), NIHSS 12 (IQR 3–21), RTPA 41.7% (see Table 1). The overall frequency of pneumonia was 10.9% (614/5652). It was present in 11.5% of the RTPA (270/2358) and 10.4% (344/3295) of the no RTPA groups. 614 (10.7%) cases. The two groups differed significantly with regards to age (p<0.001), baseline NIHSS (p<0.001), beta blockers (p<0.001), benzodiazepines (p<0.001), diabetes (p<0.001), hypertension (p<0.001) and sex (p = 0.0049).

**Table 1:** Comparisons of people receiving RTPA and not receiving RTPA in the VISTA-Acute data set.

Features	RTPA (n = 2358)	No RTPA (n = 3295)	p-value
BNIH	14 (IQR 6–22)	12 (IQR 4–20)	<0.001
Age	71 (IQR 53–89)	72 (IQR 55–89)	<0.001
Sex (%, Male)	56.8	53.2	0.0049
BB2	0.53	0.45	<0.001
BENZ2	0.35	0.29	<0.001
Diabetes	0.2	0.25	<0.001
Hypertension	0.7	0.76	<0.001
Atrial Fibrillation	0.24	0.27	0.0089
CHF	0.09	0.09	0.98
Pneumonia	0.115	0.104	0.23
Rankin ≤2	0.422	0.402	0.141
mortality	0.153	0.173	0.038

BNIH = baseline NIHSS, BENZ2 = benzodiazepine, BB2 = beta blockers, IHD = ischemic heart disease, CHF, congestive heart failure, AFIB = atrial fibrillation, RTPA = recombinant tissue plasminogen activator.

### Random forest

The AUC in the training model was 0.70 (95% CI 0.65–0.76), and in the test model was 0.67 (95% CI 0.62–0.73) and out of bag (left over data from training) error for the training data was 11.04% (calculated from left over data to estimate error in model). The Shapley value shows that low baseline stroke severity (NIHSS ≤10), younger age (≤50 years) made the largest contribution to lowering the frequency of pneumonia while absence of history of hypertension and male sex have the largest effect on pneumonia (Fig 1). In this figure, the Shapley value illustrates the ‘payout’ of the feature within the model and the direction of its impact on the classification of pneumonia. The difference between the actual and average prediction implies that the sum of the payout for prediction is 0.10. Among the medications, alteplase and beta-blockers has minimal effect on pneumonia. By contrast, absence of benzodiazepine has a greater payout on pneumonia. Feature importance illustrates the impact on the model’s error by permuting the features. Permutation of age and baseline NIHSS had the greatest effect on the model’s error, follow by gender, atrial fibrillation and benzodiazepines and beta blockers. The probability of pneumonia increased in step-wise manner above an NIHSS score of 10 (Fig 2). For age, the probability of pneumonia increased from 80 years of age onwards. We used the interaction strength of the features to illustrate the complex relationship among the features. The interaction strength (H-statistic) is strong for age and baseline stroke severity and lower for RTPA.

## Discussion

The main findings in our study are that RTPA and beta-blockers had a minimal effect on the proportion of people having pneumonia following stroke. Using Shapley value, the baseline stroke severity and age dominate the ‘payout’ for pneumonia with the probability of pneumonia lower at stroke severity less than 10 and age less than 50. This finding on stroke severity and age is consistent with other studies on stroke-associated pneumonia. Our finding on the possible role of benzodiazepines requires further evaluation in the future.

### VISTA-Acute

**Fig 3:**
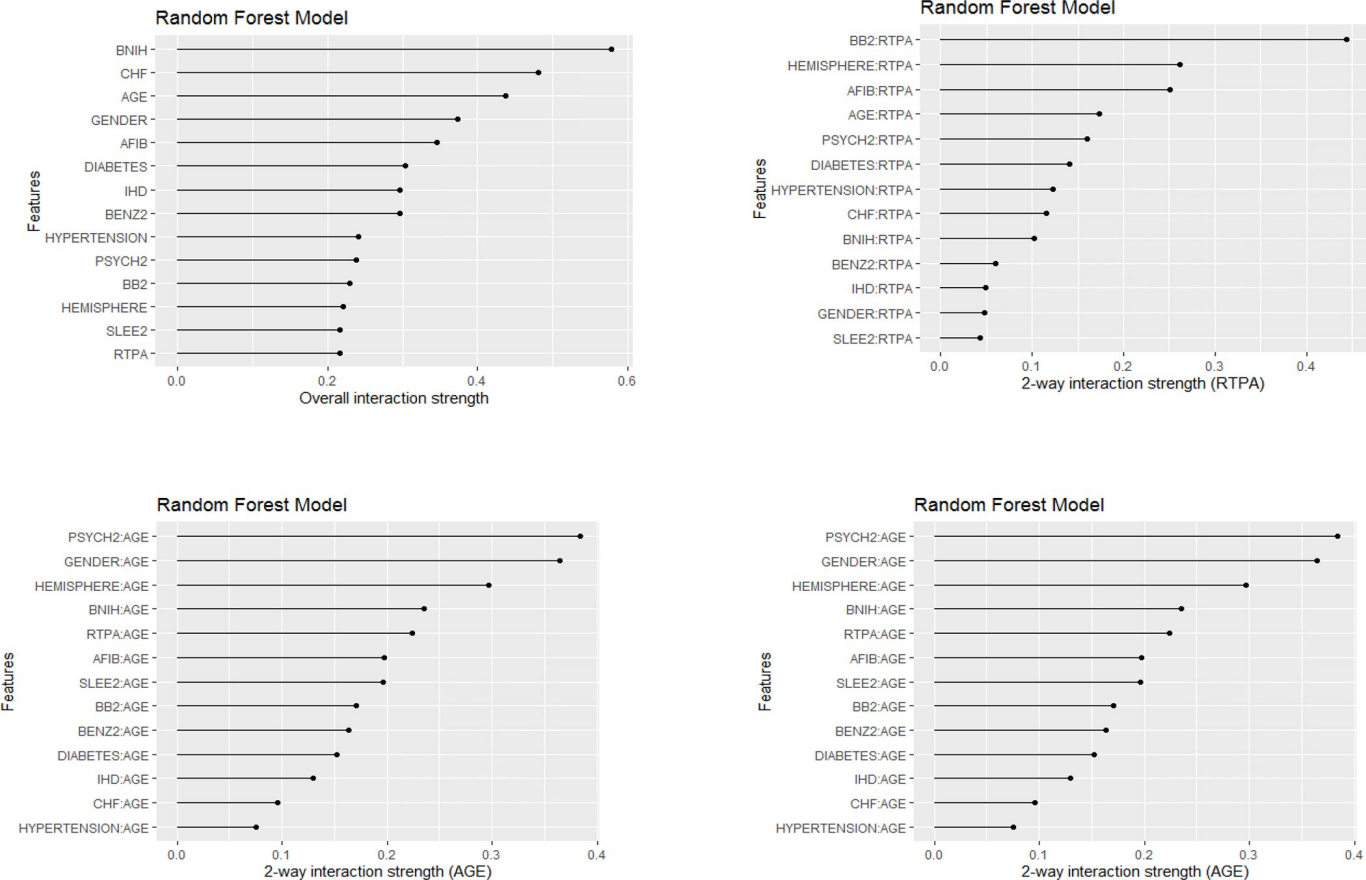
The interaction strength (H-statistic) is strong for age and baseline stroke severity and almost reach 70% of variance explained per feature. BNIH = baseline NIHSS, BENZ2 = benzodiazepine, BB2 = beta blockers, SLEE2 = benzodiazepine and non-benzodiazepine sleeping medications, IHD = ischemic heart disease, CHF, congestive heart failure, AFIB = atrial fibrillation, RTPA = recombinant tissue plasminogen activator.

The motivation for this study were the potential effect of alteplase on lowering stroke severity [19]. This finding generates the hypothesis that lower NIHSS from RTPA would be associated with lower frequency of pneumonia. However, if the effect of RTPA on pneumonia exists, it’s relatively modest as can be visualised from the model. Random forest is based on decision trees but it’s not simple to print 200 trees for the readers to understand. This is in part due to the ensemble machine learning process hinted in the introduction whereby the algorithm takes the ‘wisdom of the crowd’ and average the contribution of the covariate to the model to generate the final model. Machine learning has a reputation as a ‘black box’ and so has previously been avoided in medicine. Recently, there has been renewed interest in machine learning but publications on the topic has focussed on the outcome model rather than explanation of the model [20]. We took advantage of changes in interpretable machine learning to look inside the ‘black box’ [14]. This approach permits a range of tools such as Game Theory [13] to determine which covariates make the largest contribution to the model (see Shapley value plot in Fig 1), visualization the relationship between the covariate of interest and the predicted outcome (see ICE plot in Fig 2) [18] and exploring the plot of interaction (Fig 3). These approachs permit examination of multiple covariates whereas more traditional regression approaches have explored the effect of medications on stroke outcome evaluate each medication on its own [8,9]. This approach is different from that obtained by using logistic regression which reduce the covariates down to those with statistical significant association with the variables (age, sex, diabetes and baseline NIHSS). However, this approach does not offer the flexibility of our approach. Within the random forest model it is possible to analyse the interactions among the covariate or the effect of covariate threshold at which the risk of pneumonia increases (see Fig 2). The feature importance plot from random forest ranks diabetes as the ninth in terms of importance whereas the the logistic regression model might draw attention to the p-value and odds ratio (see Table 1).

The key findings from random forest is the importance of baseline NIHSS followed by age and sex (feature importance plot). In this plot, hypertension, heart failure and sleeping medication ranked lowest. The Shapley value plot illustrates the ‘payout’ of the feature and show the impact of the feature on the classification of pneumonia in Fig 1. The baseline NIHSS has the largest payout with the threshold being at 10. As the NIHSS increases above 10 the effect on the payout favour pneumonia. This was also the age with threshold at 50-year-old.

These threshold are shown again in the Individual Condition Expectation (ICE) curve in Fig 2. The direction of the payout for hypertension is that the absence of history of hypertension favours classification of penumonia. As the reviewer points out, this finding seems counterintuitative. It’s not clear from the data the reason for this. The interaction plot suggests strong interaction with beta-blockers (S2 Fig) but weak interaction between hypertension with age and baseline NIHSS. For example, studies have explored the use of angiotensin converting ezyme inhibitor in reduction of post-stroke pneumonia [21]. However, the data on this is still early and we would caution the interpretation until further data become available.

Our finding on the importance of NIHSS in stroke-associated pneumonia has been demonstrated by other investigators as well as data from our centre [11,22,23]. In our data stroke severity made the largest contributor to pneumonia at 72.8% whereas dysphagia only contributed 3.8% [11]. A limitation is that we did not have data on dysphagia in this clinical trial registry cohort to evaluate its importance. Investigators in the past and recently have alluded to the importance of dysphagia [23]. In analysis of data from our centre and using Shapley value regression, we showed that the proportional contribution of dysphagia to stroke associated pneumonia was small relative to NIHSS [11]. Similar to dysphagia, other important variables such as nasogastric tubes were not available [6]. One possibility is the variability in the management.

### Medications

The prior papers describing a potential effect of beta-blocker [8] on pneumonia but have not explored the effect of other medications such as benzodiazepine in the same model [9]. Our finding suggests a small effect of benzodiazepine on pneumonia. The plot shows that benzodiazepine has a larger payout that alteplase (RTPA) or beta-blockers on pneumonia. The model framed it in the negative as pneumonia was less likely to occur among patients not taking benzodiazepine. A previous analysis of the VISTA-Acute data suggested that benzodiazepine is not associated with post-stroke pneumonia [9]. However, these papers have not analyzed these medications together such as we have done here with random forest [8,9]. Our finding has support in that benzodiazepine has been associated with pneumonia in chronic phase of stroke [24]. Meta-analysis of observation studies on the use of benzodiazepine, outside the setting of stroke, suggested that its use increased the risk of pneumonia [25]. The reason for this is observation not clear. In the context of stroke, benzodiazepine may lower the level of consciousness, but the interaction plot (Fig 3) does not support this postulate. Another possibility is that benzodiazepine may be used as a sedating drug in elderly patients with delirium. Against this postulate, other sleeping aid medications and psychotropic drugs were not associated with increased risk of pneumonia in our cohort.

We do not have data on why these patients were given benzodiazepine and at which stage of the disease that the patients were given benzodiazepine. Because we had examined this drug as a class effect, we did not take dosage into consideration. Furthermore, we cannot be certain if benzodiazepine could have modified the risk of pneumonia in patients who were drowsy, had severe stroke deficit and dysphagia. The role of benzodiazepine in stroke is interesting. Previously, benzodiazepine had been explored as a drug to reduce stroke disability via its action on gamma-amino-butyric acid (GABA) [26]. Others have also proposed that benzodiazepine be used to diagnosed transient ischemic attack via its ability to reintroduce neurological deficit [27]. It is likely that further works need to be done in this area.

### Limitations

A potential issue with using VISTA-Acute data is that the data represents patients randomized to clinical trials and thus the cohort may not reflect the breadth of patients usually seen in hospitals. Those excluded from clinical trials can include individuals with the most severe strokes or have multiple comorbidities. Furthermore, we have data on onset to treatment with neuroprotection drug but do not have raw data on time of onset or time of hospital arrival. Such data can help to determine if pneumonia was related to stroke occurring during sleep. The diagnosis of post-stroke pneumonia was based on reports of serious adverse events [8]. This approach has been used by several different groups evaluating diffferent aspects of pneumonia from VISTA-Acute [8,9,28]. This approach is different from the clinical approach and thus has the potential to under report the number of cases. We were careful in this analysis by having a set dictionary of terms to search for the diagnosis of pneumonia from SAE log. A potential approach is to use ICD-10 codes for the diagnosis of pneumonia but this route was not available given that the data came from clinical trial registry and which contains de-identified data from multiple trials. In this analysis, data on other parameters such as blood test results could not be used due to large proportion of missing values. On the question of thrombectomy, we have not measured this as the trials gathered in this dataset were neuroprotection trials and not RTPA or thrombectomy trials. Since the data were from the pre-thrombectomy era, it is likely that the number of such cases were very small. On the issue of beta-blocker and pneumonia, we found that the proportional contribution of it to the random forest model was low (Fig 1). Our finding appears on the surface different to previous analyis which had used Poisson regression but this might relate to methodological approach [8]. Those investigators had found risk reduction with beta-blockers but with wide confidence interval [8].

## Conclusion

Ensemble machine learning method showed the importance of baseline stroke severity and age in model of post stroke pneumonia. There is a possibility that benzodiazepine may contribute but this needs to be evaluated outside of VISTA type of dataset. More research should be directed at the effect of other medications on stroke outcome. We were not able to confirm the importance of RTPA or beta blocker in development of pneumonia in VISTA-Acute. We will approach other investigators regarding availability of data from thrombolysis trials to perform this analysis prior to concluding the role of RTPA on pneumonia.

## Supporting information

S1 Fig The error rate decreases with more trees added. (DOCX)

S2 Fig The interaction strength (H-statistic) is strong for age and baseline stroke severity and almost reach 70% of variance explained per feature. BNIH = baseline NIHSS, BENZ2 = benzodiazepine, BB2 = beta blockers, SLEE2 = benzodiazepine and non-benzodiazepine sleeping medications, IHD = ischemic heart disease, CHF, congestive heart failure, AFIB = atrial fibrillation, RTPA = recombinant tissue plasminogen activator.
(DOCX)

S1 Appendix (DOCX)

## References

[1] Collaborators GBDS. Global, regional, and national burden of stroke and its risk factors, 1990–2019: a systematic analysis for the Global Burden of Disease Study 2019. Lancet Neurol. 2021;20(10):795–820. Epub 2021/09/07. doi: 10.1016/S1474-4422(21)00252-0 ; PubMed Central PMCID: PMC8443449. 34487721 PMC8443449

[2] Westendorp WF, Nederkoorn PJ, Vermeij JD, Dijkgraaf MG, van de Beek D. Post-stroke infection: a systematic review and meta-analysis. BMC Neurol. 2011;11:110. doi: 10.1186/1471-2377-11-110 ; PubMed Central PMCID: PMC3185266. 21933425 PMC3185266

[3] Bray BD, Smith CJ, Cloud GC, Enderby P, James M, Paley L, et al. The association between delays in screening for and assessing dysphagia after acute stroke, and the risk of stroke-associated pneumonia. J Neurol Neurosurg Psychiatry. 2017;88(1):25–30. doi: 10.1136/jnnp-2016-313356 . 27298147

[4] Kalra L, Irshad S, Hodsoll J, Simpson M, Gulliford M, Smithard D, et al. Prophylactic antibiotics after acute stroke for reducing pneumonia in patients with dysphagia (STROKE-INF): a prospective, cluster-randomised, open-label, masked endpoint, controlled clinical trial. Lancet. 2015;386(10006):1835–44. doi: 10.1016/S0140-6736(15)00126-9 . 26343840

[5] Langdon PC, Lee AH, Binns CW. High incidence of respiratory infections in ’nil by mouth’ tube-fed acute ischemic stroke patients. Neuroepidemiology. 2009;32(2):107–13. doi: 10.1159/000177036 . 19039243

[6] Dziewas R, Ritter M, Schilling M, Konrad C, Oelenberg S, Nabavi DG, et al. Pneumonia in acute stroke patients fed by nasogastric tube. J Neurol Neurosurg Psychiatry. 2004;75(6):852–6. doi: 10.1136/jnnp.2003.019075 ; PubMed Central PMCID: PMC1739077. 15145999 PMC1739077

[7] Warusevitane A, Karunatilake D, Sim J, Lally F, Roffe C. Safety and effect of metoclopramide to prevent pneumonia in patients with stroke fed via nasogastric tubes trial. Stroke. 2015;46(2):454–60. doi: 10.1161/STROKEAHA.114.006639 . 25516196

[8] Sykora M, Siarnik P, Diedler J, Collaborators VA. beta-Blockers, Pneumonia, and Outcome After Ischemic Stroke: Evidence From Virtual International Stroke Trials Archive. Stroke. 2015;46(5):1269–74. doi: 10.1161/STROKEAHA.114.008260 . 25899243

[9] Frank B, Fulton RL, Lees KR, Sanders RD, Collaborators V. Impact of benzodiazepines on functional outcome and occurrence of pneumonia in stroke: evidence from VISTA. Int J Stroke. 2014;9(7):890–4. doi: 10.1111/ijs.12148 . 24148353

[10] Zhang SR, Phan TG, Sobey CG. Targeting the Immune System for Ischemic Stroke. Trends in pharmacological sciences. 2021;42(2):96–105. Epub 2020/12/21. doi: 10.1016/j.tips.2020.11.010 . 33341247

[11] Phan TG, Kooblal T, Matley C, Singhal S, Clissold B, Ly J, et al. Stroke Severity Versus Dysphagia Screen as Driver for Post-stroke Pneumonia. Frontiers in neurology. 2019;10:16. doi: 10.3389/fneur.2019.00016 ; PubMed Central PMCID: PMC6361825. 30761063 PMC6361825

[12] Friedman J, Popescu BE. Predictive learning via rule ensembles. The Annals of Applied Statistics. 2008;2:916–54. doi: 10.1214/07-AOAS148

[13] Shapley LS. A Value for N-Person Games. Contributions to the Theory of Games 1953. p. 307–17.

[14] Molnar C. Interpretable Machine Learning: A Guide for Making Black Box Models Explainable https://christophm.github.io/interpretable-ml-book/2018 [cited 2018 17/7/2018]. Available from: https://christophm.github.io/interpretable-ml-book/.

[15] Ali M, Bath P, Brady M, Davis S, Diener HC, Donnan G, et al. Development, expansion, and use of a stroke clinical trials resource for novel exploratory analyses. Int J Stroke. 2012;7(2):133–8. Epub 2012/01/24. doi: 10.1111/j.1747-4949.2011.00735.x . 22264365

[16] Barber PA, Demchuk AM, Zhang J, Buchan AM. Validity and reliability of a quantitative computed tomography score in predicting outcome of hyperacute stroke before thrombolytic therapy. ASPECTS Study Group. Alberta Stroke Programme Early CT Score. Lancet. 2000;355:1670–4. doi: 10.1016/s0140-6736(00)02237-6 . 10905241

[17] Štrumbelj E, Kononenko I. Explaining prediction models and individual predictions with feature contributions. Knowl Inf Syst (2014) 41:647–665. 2014;41:647–65. doi: 10.1007/s10115-013-0679-x

[18] Goldstein A, Kapelner A, Bleich J, Pitkin E. Peeking Inside the Black Box: Visualizing Statistical Learning With Plots of Individual Conditional Expectation. Journal of Computational and Graphical Statistics. 2015;(1):44–65. 10.1080/10618600.2014.907095.

[19] Ma H, Campbell BCV, Parsons MW, Churilov L, Levi CR, Hsu C, et al. Thrombolysis Guided by Perfusion Imaging up to 9 Hours after Onset of Stroke. N Engl J Med. 2019;380(19):1795–803. doi: 10.1056/NEJMoa1813046 . 31067369

[20] Heo J, Yoon JG, Park H, Kim YD, Nam HS, Heo JH. Machine Learning-Based Model for Prediction of Outcomes in Acute Stroke. Stroke. 2019;50(5):1263–5. Epub 2019/03/21. doi: 10.1161/STROKEAHA.118.024293 . 30890116

[21] Shinohara Y, Origasa H. Post-stroke pneumonia prevention by angiotensin-converting enzyme inhibitors: results of a meta-analysis of five studies in Asians. Adv Ther. 2012;29(10):900–12. Epub 2012/09/18. doi: 10.1007/s12325-012-0049-1 . 22983755

[22] Westendorp WF, Vermeij JD, Hilkens NA, Brouwer MC, Algra A, van der Worp HB, et al. Development and internal validation of a prediction rule for post-stroke infection and post-stroke pneumonia in acute stroke patients. Eur Stroke J. 2018;3(2):136–44. Epub 2018/06/15. doi: 10.1177/2396987318764519 ; PubMed Central PMCID: PMC5992742. 29900413 PMC5992742

[23] Jannini TB, Ruggiero M, Vigano A, Comanducci A, Maestrini I, Giuliani G, et al. The role of the Sapienza GLObal Bedside Evaluation of Swallowing after Stroke (GLOBE-3S) in the prevention of stroke-associated pneumonia (SAP). Neurol Sci. 2022;43(2):1167–76. Epub 2021/07/17. doi: 10.1007/s10072-021-05449-y ; PubMed Central PMCID: PMC8789723. 34269936 PMC8789723

[24] Lin SM, Yang SH, Liang CC, Huang HK, Loh CH. Association between benzodiazepine use and risks of chronic-onset poststroke pneumonia: a population-based cohort study. BMJ open. 2019;9(1):e024180. Epub 2019/02/21. doi: 10.1136/bmjopen-2018-024180 ; PubMed Central PMCID: PMC6347861. 30782728 PMC6347861

[25] Sun GQ, Zhang L, Zhang LN, Wu Z, Hu DF. Benzodiazepines or related drugs and risk of pneumonia: A systematic review and meta-analysis. Int J Geriatr Psychiatry. 2019;34(4):513–21. Epub 2019/01/10. doi: 10.1002/gps.5048 . 30623504

[26] Lodder J, van Raak L, Hilton A, Hardy E, Kessels A, Group ES. Diazepam to improve acute stroke outcome: results of the early GABA-Ergic activation study in stroke trial. a randomized double-blind placebo-controlled trial. Cerebrovasc Dis. 2006;21(1–2):120–7. Epub 2005/12/13. doi: 10.1159/000090210 . 16340187

[27] Lazar RM, Fitzsimmons BF, Marshall RS, Mohr JP, Berman MF. Midazolam challenge reinduces neurological deficits after transient ischemic attack. Stroke. 2003;34(3):794–6. doi: 10.1161/01.STR.0000056540.04159.F3 . 12624310

[28] de Jonge JC, van de Beek D, Lyden P, Brady MC, Bath PM, van der Worp HB. Temporal Profile of Pneumonia After Stroke. Stroke. 2021:STROKEAHA120032787. Epub 2021/09/15. doi: 10.1161/STROKEAHA.120.032787 . 34517764 PMC8700305

