# Supplementary figures and images for: Effect of alteplase, benzodiazepines and beta-blocker on post-stroke pneumonia: Exploration of VISTA-Acute
Source: PLoS One. 2023 May 1;18(5):e0281617. doi: 10.1371/journal.pone.0281617 (PMC10150972; doi:10.1371/journal.pone.0281617)

Supplementary Figure 1: Estimation of number of trees needed
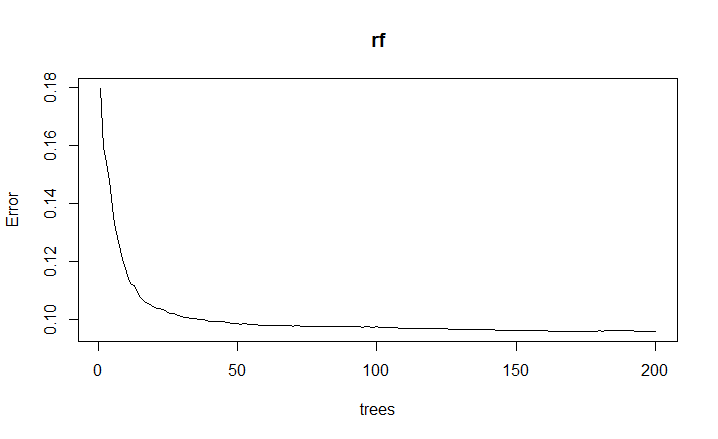

Supplement: S1 Fig — (DOCX) [file pone.0281617.s001.docx]

Supplementary Figure 2


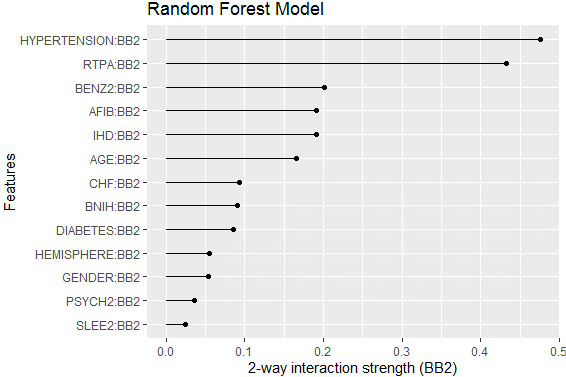

Supplement: S2 Fig — BNIH = baseline NIHSS, BENZ2 = benzodiazepine, BB2 = beta blockers, SLEE2 = benzodiazepine and non-benzodiazepine sleeping medications, IHD = ischemic heart disease, CHF, congestive heart failure, AFIB = atrial fibrillation, RTPA = recombinant tissue plasminogen activator. (DOCX) [file pone.0281617.s002.docx]
