# Supplementary material for: Effect of alteplase, benzodiazepines and beta-blocker on post-stroke pneumonia: Exploration of VISTA-Acute
Source: PLoS One. 2023 May 1;18(5):e0281617. doi: 10.1371/journal.pone.0281617 (PMC10150972; doi:10.1371/journal.pone.0281617)
Supplement: S1 Appendix — (DOCX) [file pone.0281617.s003.docx]

Appendix

*VISTA-Acute Steering Committee: Kennedy R. Lees (Chair), A. Alexandrov, P.M. Bath, E. Berge, E. Bluhmki, N. Bornstein, C. Chen, L. Claesson, S.M. Davis, H-C. Diener, G. Donnan, M. Fisher, M. Ginsberg, B. Gregson, J .Grotta, W. Hacke, M.G. Hennerici, M. Hommel, M. Kaste, P. Lyden, J. Marler, K. Muir, R. Sacco, A. Shuaib, P. Teal, N. Venketasubramanian, N.G. Wahlgren, and S. Warach.

Kennedy R Lees, [kennedy.Lees@glasgow.ac.uk](mailto:kennedy.Lees@glasgow.ac.uk) (Chair), School of Medicine, Dentistry, and Nursing, University of Glasgow, Glasgow, United Kingdom

Andrei Alexandrov [avalexandrov@att.net](mailto:avalexandrov@att.net), Department of Neurology, The University of Tennessee Health Science Center, 855 Monroe Avenue, Suite 415, Memphis, TN 38163, USA

Phil M Bath, [philip.bath@nottingham.ac.uk](mailto:philip.bath@nottingham.ac.uk), Stroke Trials Unit, University of Nottingham, UK5

Eivind Berge [eivind.berge@medisin.uio.no](mailto:eivind.berge@medisin.uio.no), Department of Internal Medicine and Cardiology, Oslo University Hospital, Oslo, Norway

Natan Bornstein [natan.bornstein@gmail.com](mailto:natan.bornstein@gmail.com), Department of Neurology, Tel-Aviv Sourasky Medical Center, Tel-Aviv, Israel; Sackler Faculty of Medicine, Tel-Aviv University, Tel-Aviv, Israel

Christopher Chen [cplhchen@yahoo.com.sg](mailto:cplhchen@yahoo.com.sg), Memory Aging &amp; Cognition Centre, Yong Loo Lin School of Medicine, National University of Singapore, Singapore

L Claesson [lisbeth.claesson@neuro.gu.se](mailto:lisbeth.claesson@neuro.gu.se), Institute of Neuroscience and Physiology, Sahlgrenska Academy at the University of Gothenburg, Sweden.

Stephen.M. Davis, [stephen.davis@mh.org.au](mailto:stephen.davis@mh.org.au), Departments of Medicine and Neurology, Melbourne Brain Centre at the Royal Melbourne Hospital, The University of Melbourne, VIC, Australia

Hans-Christoph Diener [h.diener@uni-essen.de](mailto:h.diener@uni-essen.de), Institute for Medical Informatics, Biometry and Epidemiology, Medical Faculty, University Duisburg-Essen, Essen, Germany.

Geoffrey Donnan, [gdonnan@unimelb.edu.au](mailto:gdonnan@unimelb.edu.au), Departments of Medicine and Neurology, Melbourne Brain Centre at the Royal Melbourne Hospital, The University of Melbourne, VIC, Australia

Marc. Fisher, [mfisher5@bidmc.harvard.edu](mailto:mfisher5@bidmc.harvard.edu), Department of Neurology, Beth Israel Deaconess Medical Center, Harvard Medical School' Boston' MA, USA

Myron. Ginsberg, [mginsberg@med.miami.edu](mailto:mginsberg@med.miami.edu), Department of Neurology, University of Miami Miller School of Medicine, Clinical Research Center, Room 1331, 1120 NW 14th Street, Miami, FL 33136, USA.

Barbara. Gregson, [ku.ca.lcn@nosgerg.arabrab](mailto:ku.ca.lcn@nosgerg.arabrab), Neurosurgical Trials Group, Institute of Neuroscience, Newcastle University, Wolfson Research Centre, Campus for Ageing and Vitality, Westgate Rd, Newcastle upon Tyne, NE4 5PL, UK

James .Grotta, [James.C.Grotta@uth.tmc.edu](mailto:James.C.Grotta@uth.tmc.edu), Clinical Innovation and Research Institute, Memorial Hermann Hospital, Houston, TX, United States.

Werner Hacke, [werner.hacke@me.com](mailto:werner.hacke@me.com), Department of Neurology, Universität Heidelberg, Heidelberg, Germany

Michael .G. Hennerici, [hennerici@neuro.ma.uni-heidelberg.de](mailto:hennerici@neuro.ma.uni-heidelberg.de), Department of Neurology, Universität Heidelberg, Germany

Marc Hommel, [rf.elbonerg-fju@lemmohm](mailto:rf.elbonerg-fju@lemmohm), INSERM CIC 003, CHU - UJ Fourier, BP 217X 38043 Cedex, Grenoble, France.

Markku. Kaste, [markku.kaste@hus.fi](mailto:markku.kaste@hus.fi), HUS Neurocenter, University of Helsinki, Finland

Patrick Lyden, [Patrick.Lyden@cshs.org](mailto:Patrick.Lyden@cshs.org), Zilkha Neurogenetics Institute, Keck School of Medicine,

Room 245, ZNI, 1501 San Pablo Street,Los Angeles, CA 90089-2821

J. Marler, [john.marler@fda.hhs.gov](mailto:john.marler@fda.hhs.gov), Division of Neurology Products, CDER, US Food & Drug Administration, Silver Spring, MD 20993-0002, USA.

Keith. Muir, [Keith.Muir@glasgow.ac.uk](mailto:Keith.Muir@glasgow.ac.uk), University of Glasgow, Queen Elizabeth University Hospital, Glasgow G51 4TF

Ralph. Sacco, [rsacco@med.miami.edu](mailto:rsacco@med.miami.edu), University of Miami, FL, USA

Ashfaq. Shuaib, [ashfaq.shuaib@ualberta.ca](mailto:ashfaq.shuaib@ualberta.ca), Department of Medicine (Neurology), University of Alberta, Edmonton, Canada

Philip. Teal, Division of Neurology, University of British Columbia, Vancouver, BC,

Nils Wahlgren [nils.wahlgren@karolinska.se](mailto:nils.wahlgren@karolinska.se), Department of Neurosciences, Karolinska Institute, Sweden

N. Venketasubramanian , Raffles Neuroscience Centre, Raffles Hospital, Singapore, Singapore.

Steve Warach [SWarach@seton.org](mailto:SWarach@seton.org), Department of Neurology, Dell Medical School, University of Texas at Austin
